# Gender-Affirming Treatment of Gender Dysphoria in Youth: A Perfect Storm Environment for the Placebo Effect—The Implications for Research and Clinical Practice

**DOI:** 10.1007/s10508-022-02472-8

**Published:** 2022-11-14

**Authors:** Alison Clayton

**Affiliations:** School of Historical and Philosophical Studies, University of Melbourne, Parkville, 3010 Australia

## Introduction

In the last decade, there has been a rapid increase in the numbers of young people with gender dysphoria (GD youth) presenting to health services (Kaltiala et al., 2020). There has also been a marked change in the treatment approach. The previous “common practice” of providing psychosocial care only to those under 18 or 21 years (Smith et al., 2001) has largely been replaced by the gender affirmative treatment approach (GAT), which for adolescents includes hormonal and surgical interventions (Coleman et al., 2022). However, as a recent review concluded, evidence on the appropriate management of youth with gender incongruence and dysphoria is inconclusive and has major knowledge gaps (Cass, 2022). Previous papers have discussed that the weaknesses of the studies investigating the efficacy of GAT for GD youth mean they are at high risk of bias and confounding and, thus, provide very low certainty evidence (Clayton, 2022a, b; Levine et al., 2022). To date, however, there has been little discussion of the inability of these studies to differentiate specific treatment effects from placebo effects. Of note, the term “placebo effect” is no longer used to just simply refer to the clinical response following inert medication; rather, it describes the beneficial effects attributable to the brain-mind responses evoked by the treatment context rather than the specific intervention (Wager & Atlas, 2015). This Letter argues that the current treatment approach for GD youth presents a perfect storm environment for the placebo effect. This raises complex clinical and research issues that require attention and debate.

## A Brief Introduction to the Contemporary Concept of the Placebo Effect

The term “placebo effect” can be used variably by different authors. As recently defined in a consensus statement, placebo (beneficial) and nocebo (deleterious) effects occur in clinical or research contexts and are due to psychobiological mechanisms evoked by the treatment (or research) context rather than any specific effect of the intervention. Importantly, placebo and nocebo effects not only occur during the prescription of placebo (inert) pills, but they can also substantially modulate the efficacy and tolerability of active medical treatments (Evers et al., 2018).

The therapeutic ritual, the encounter between a sick person and a clinician, is a powerful psychosocial event. Clinicians, particularly physicians, are our society’s designated healers and their prestige, status, and authority help engender patients’ trust and expectations of relief from suffering (Benedetti, 2021a). Positive clinician–patient interactions are associated with decreased anxiety and increased hope. Complex neurobiological mechanisms are implicated in the placebo effect, including release of neurotransmitters (e.g., endorphins, cannabinoids, dopamine, and oxytocin) and activation of specific areas of the brain (e.g., the prefrontal cortex, anterior insula, rostral anterior cingulate cortex, and the amygdala) (Colloca & Barsky, 2020; Kaptchuk & Miller, 2015). These changes are associated with an increased sense of well-being. They also impact on cardiovascular, respiratory, immune, and endocrine functioning, all of which may contribute to patients’ clinical improvement (Enck et al., 2013; Wager & Atlas, 2015).

Several unconscious psychological mechanisms, including classical conditioning and social learning, play a role in the placebo effect (Benedetti, 2021a). In clinical trials, where patients communicate with each other, a process of social observational learning may be associated with emotional contagion and, thus, placebo and nocebo effects (Benedetti, 2013). The media and social media may also foster these effects and contribute to the dissemination of symptoms and illness throughout the general population (Colloca & Barsky, 2020).

Expectation of outcome is a principal mechanism of the placebo effect and anything that increases patients’ expectations is potentially capable of boosting placebo effects (Evers et al., 2018). Although research has demonstrated that changes in physiological parameters may occur following placebo administration (Wager & Atlas, 2015), these response expectations have been particularly noted in patient-reported outcomes, such as anxiety, pain, life satisfaction, and mood. Expectations and cognitive readjustment can lead to behavioral changes, such as resuming normal daily activities, which can be observer rated. Physicians’ status, whether through the general position given to them in society or through individual personality factors, may contribute to such expectations of benefit. This type of phenomenon has sometimes been termed prestige suggestion. The “Hawthorne effect” describes the phenomenon where clinical trial patients’ improvements may occur because they are being observed and given special attention. A patient who is part of a study, receiving special attention, and with motivated clinicians, who are invested in the benefits of the treatment under study, is likely to have higher expectations of therapeutic benefits (Benedetti, 2021a).

Placebo-induced improvements are real and can be robust and long lasting (Benedetti, 2021b; Wager & Atlas, 2015). Individual patient factors, such as personality and genetics, may be associated with placebo responsiveness (Benedetti, 2021a). The particular illness is also relevant. For example, although placebo treatment can impact symptoms of cancer, there is no evidence that placebos can shrink tumors (Benedetti, 2021b; Kaptchuk & Miller, 2015). However, there is evidence that placebos can act as long-lasting and effective treatments for depression and various pain conditions, such as migraine and osteoarthritic knee pain (Kam-Hansen et al., 2014; Kirsch, 2019; Previtali et al., 2021). Further, some research suggests adherence to placebo medication, particularly in cardiac disease, may be associated with reduced mortality (Wager & Atlas, 2015).

## The Research Setting versus the Clinical Practice Setting

Research into new medical treatments aims to control for placebo effects, and this helps ensure true evaluation of the treatment’s efficacy (Enck et al., 2013). The double-blind randomized controlled trial (DBRCT), although not perfect, is the current gold standard for determining the efficacy and safety of a treatment. The DBRCT study design evolved over several centuries and became widely accepted practice in the mid-twentieth century (Lilienfeld, 1982). Of note, the term “blind” is thought to have originated in eighteenth-century France when blindfolds were used to disprove Anton Mesmer’s “animal magnetism” theory and the mesmerism craze of that era (Kaptchuk, 1998). Well-designed DBRCTs minimize the impact of bias, confounding and placebo effects on findings and are the best type of study for determining whether there is a causal relationship between an intervention and an effect (Enck et al., 2013; Kabisch et al., 2011).

The reader may wonder about this requirement of differentiating placebo effects from the specific effects of an intervention and ask: If the patient improves, does it really matter why? Yes, it does, particularly for treatments that have significant risk of adverse effects. There are also broader problems raised by relying on the placebo effect. Consider prescribing antibiotics for viral infections. The patient may experience clinical benefit through a placebo effect. However, not only may some patients experience serious adverse drug reactions, but the health of the whole population is imperiled by the problem of antibiotic resistance (Llor & Bjerrum, 2014). Furthermore, informed consent is an ethical pillar of modern medicine and requires clinician honesty and transparency. Clinicians deceptively utilizing placebo treatments do not meet this requirement (Barnhill, 2012; Kaldjian & Pilkington, 2021). A medical profession that does little to distinguish placebo effects from specific treatment effects risks becoming little different from pseudoscience and the quackery that dominated medicine of past times, with likely resulting decline in public trust and deterioration in patient outcomes (Benedetti, 2021a).

Ideally, in evidence-based medicine, a new treatment undergoes rigorous research and has reasonable evidence of benefit prior to being introduced as routine treatment (although ongoing further research often continues). Clinicians can then reasonably harness and enhance the placebo effect to improve outcomes (Enck et al., 2013). A placebo effect enhancing clinical setting, in which warm and empathic clinicians provide supportive and attentive health care, creates a “therapeutic bias” in patients, giving them hope and expectation of improvement. This is “legitimate” so long as it is done without deception and in a manner consistent with informed consent, trust, and transparency (Kaptchuk & Miller, 2015).

This ideal of clinical interventions having solid evidence of efficacy before being introduced as routine practice is not always a reality. Sometimes, it is more of a situation where the “cart” of clinical practice precedes the “horse” of rigorous research evidence. Then, this catch-up research may be undertaken in a placebo effect-enhancing clinical environment, rather than a placebo effect-controlled research environment. Such situations, especially when DBRCT are not possible, present the researcher and clinician with complex research and clinical conundrums. Some of these will now be explored using the example of the treatment of youth with gender dysphoria.

## A Brief Introduction to the Gender-Affirming Treatment Model for Children and Adolescents with Gender Dysphoria

Gender dysphoria is a term used to describe the distress that is frequently felt by people whose sense of gender is incongruent with their natal sex (these people may also self-identify as transgender) and if the dysphoria is intense and persistent, alongside several other features, a DSM-5 diagnosis of gender dysphoria may be made (American Psychiatric Association, 2013). There has been a sharp rise in the numbers of children and adolescents identifying as transgender and being diagnosed with gender dysphoria (Kaltiala et al., 2020; Tollit et al., 2021; Wood et al., 2013). Many are natal sex females presenting in adolescence, and many have neurodevelopmental and psychiatric disorders (Kaltiala-Heino et al., 2018; Tollit et al., 2021; Zucker, 2019). International guidelines and child and adolescent gender clinics (CAGCs) commonly endorse a gender affirmative treatment approach (GAT) (Coleman et al., 2022; Hembree et al., 2017; Olson-Kennedy et al., 2019; Telfer et al., 2018). Key components of GAT include affirmation of a youth’s stated gender identity, facilitation of early childhood social transition, provision of puberty blockers to prevent the pubertal changes consistent with natal sex, and use of cross-sex hormones (CSH) and surgical interventions to align physical characteristics with gender identity (Ehrensaft, 2017; Rosenthal, 2021). This Letter’s discussion focuses primarily on the medical (puberty blockers and cross-sex hormones) and surgical elements of GAT.

GAT can achieve some of the desired masculine or feminine appearance outcomes, but the main arguments used to support the use of these treatments in GD youth are that they improve short- and long-term mental health and quality-of-life outcomes. However, this claim is only underpinned by low-quality (mostly short-term, uncontrolled, observational) studies, which provide very low certainty evidence, complemented by expert opinion (Clayton, 2022a; Hembree et al., 2017; NICE, [Bibr CR74][Bibr CR73]; Rosenthal, 2021). No randomized controlled trials (RCTs), including none using the previous treatment approach as a comparative, have been undertaken. This low-quality evidence for the efficacy of GAT is of particular concern given the potential risks associated with GAT.

## Risks of Gender-Affirming Medical and Surgical Treatments

Impaired fertility is a risk of cross-sex hormones, and the extent of reversibility of this is unclear (Cheng et al., 2019; Hembree et al., 2017). If puberty blockers are commenced in early puberty and followed by cross-sex hormones, there are no proven methods of fertility preservation (Bangalore Krishna et al., 2019). Surgeries, such as gonadectomies and most genital surgeries, will result in permanent sterility. These impaired fertility and sterility outcomes are important because, firstly, as Cheng et al. (2019) reported, the widespread assumption that many transgender people do not want to have biological children is not supported by several recent studies. Secondly, children as young as ten, who do not have capacity for informed consent, are starting a treatment course that will likely render them infertile or sterile and this raises complex bioethical issues (Baron & Dierckxsens, 2021).

Other adverse effects of GAT are based on a more uncertain evidence base. I provide a brief outline of some of the areas of concern. Cross-sex hormones are associated with cardiovascular health risks, such as thromboembolic, coronary artery, and cerebrovascular diseases (Hembree et al., 2017; Irwig, 2018). Cross-sex hormones may also increase the risk of certain cancers (Hembree et al., 2017; Mueller & Gooren, 2008). Puberty blockers may have negative impact on bone mineral density, which may not be fully reversible, with an associated risk of osteoporosis and fractures (Biggs, 2021; Hembree et al., 2017). Recently, findings from animal studies have increased concerns that puberty blockers may negatively and irreversibly impact brain development due to critical time-windows of brain development. In one study on rams, long-term spatial memory deficits induced by use of puberty blockers in the peripubertal period were found to persist into adulthood (Hough et al., 2017). For those young patients who undertake surgery, there are also the risks of surgical complications (Akhavan et al., 2021). One understudied outcome of mastectomies, for those who later want to and can become pregnant, is the grief about inability to breast feed.

Puberty blockers, cross-sex hormones and genital surgery also pose risks to sexual function, particularly the physiological capacity for arousal and orgasm. It is important to be aware there is a dearth of research studying the impact of GAT on GD youth’s sexual function, but I provide a brief discussion of this important topic. Estrogen use in transwomen is associated with decreased sexual desire and erectile dysfunction and testosterone for transmen may lead to vaginal atrophy and dyspareunia (Hembree et al., 2017). It seems widely assumed that testosterone simply improves transmen’s sexual functioning. However, placebo-controlled studies from the non-transgender population indicate the situation is likely more complex. For example, studies indicate that testosterone may impact female sexual desire in a bell-shape curve manner, and at high levels may have no benefit or even have negative impact on sexual function (Krapf & Simon, 2017; Reed et al., 2016). Also of note, in medical conditions that are associated with high testosterone levels, such as polycystic ovarian syndrome, impaired sexual function (e.g., arousal, lubrication, sexual satisfaction, and orgasm) has been reported (Pastoor et al., 2018).

Recently, surgeon and WPATH president-elect, Marci Bowers, raised concern that puberty blockers given at the earliest stages of puberty to birth sex males, followed by cross-sex hormones and then surgery, might adversely impact orgasm capacity because of the lack of genital tissue development (Ley, 2021). One study has reported that some young adults, who had received puberty blockers, cross-sex hormones and laparoscopic intestinal vaginoplasty, self-reported orgasmic capacity (Bouman et al., 2016). However, this finding does not negate Bower’s concerns, as it did not make any assessment of the correlation between Tanner stage at initiation of puberty blockers with orgasm outcome. Of note, some of the patients in the study were over the age of 18 at start of GAT. Further, its findings do not apply to those undergoing penile skin inversion vaginoplasty. Importantly, Bouman et al. found that 32% of their participants self-reported being sexually inactive and only 52% reported having had neovaginal penetrative sex more than once. A recent literature review on sexual outcomes in adults post-vaginoplasty noted the paucity of high-quality evidence but reported that “up to 29% of patients may be diagnosed with a sexual dysfunction due to associated distress with a sexual function disturbance” (Schardein & Nikolavsky, 2022). Another recent systematic review of vaginoplasty reported an overall 24% post-surgery rate of inability to achieve orgasm (Bustos et al., 2021).

Coleman et al. (2022) claimed that “longitudinal data exists to demonstrate improvement in romantic and sexual satisfaction for adolescents receiving puberty suppression, hormone treatment and surgery.” However, the supporting citation requires scrutiny. Bungener et al. (2020) was a cross-sectional study of 113 young adults, 66% of whom were transmen (most who had undergone mastectomy and gonadectomy, not genital surgery). For its claims of post-surgery increases in sexual experience, it relied on recall of pre-surgical experiences. This means it is at high risk of recall bias, especially given surgery was undertaken up to 5 years (mean 1.5 years) prior to assessment. Further, it focused on sexual experiences, which might naturally be expected to increase as adolescents enter young adulthood, and there was no evaluation of sexual function domains, such as arousal, orgasm, or pain. The study did report current sexual satisfaction but failed to compare this to pre-surgical functioning (or to the Dutch peer comparison group). Thus, it is unable to demonstrate whether sexual satisfaction improved following GAT. On the three questions about sexual satisfaction (frequency, how good sex feels, and sex life in general), 59 to 73% were reportedly moderately to very satisfied. This would appear to mean that 27 to 41% were not satisfied, which is a sizeable minority. Importantly, these sexual satisfaction questions had an approximately 45% missing data rate—an issue not discussed by the authors. This means the authors’ conclusion that the majority was satisfied with their sex life is at high risk of bias. Of additional note, at the post-surgical assessment time these young transgender adults were significantly less sexually experienced than their Dutch peers. Thus, in sum, this study provides little reassurance about the sexual function outcomes of GAT in GD youth.

Lastly, in terms of risks, there are increasing reports of discontinuation of hormone treatments, regret and detransition in young people who have received GAT (Boyd et al., 2022; Hall et al., 2021; Littman, 2021; Vandenbussche, 2022). Two recent studies have relied on pharmaceutical prescription records, both using 2018 as the end date of data collection (Roberts et al., 2022; van der Loos et al., 2022). Their reported rates of discontinuation varied widely. For the US cohort, Roberts et al. (2022) reported, for those who had started CSH treatment before age 18, a 4-year CSH discontinuation rate of 25%. For the Dutch cohort, van der Loos et al. (2022) reported on CSH discontinuation rates in adolescents, evaluated according to the “meticulous” Dutch protocol, who had commenced puberty blockers before age 18. People “assigned female at birth” had a CSH discontinuation rate of 1% at a median of 2.3-years follow-up, and those “assigned male at birth” had a 4% discontinuation rate at median 3.5-years follow-up. Previous research from this Dutch group has indicated that average time to detransition was over 10 years (Wiepjes et al., 2018). Thus, given the van der Loos et al. (2022) study’s short median follow-up time and young follow-up age (median 19.2 for people “assigned female at birth” and 20.2 for “assigned male at birth”), it seems likely that these discontinuation rates will increase over time. It is also concerning to note that 75% of the Dutch youth who discontinued CSH had undergone gonadectomies, but at follow-up they were receiving neither CSH nor sex hormones consistent with their birth sex.

## Ongoing Research

Currently, several large long-term observational studies are underway which involve collecting and analyzing data on patients receiving routine GAT at CAGCs (Olson-Kennedy et al., 2019; Tollit et al., 2019). The aims of these studies are to provide the urgently needed rigorous empirical data to bolster the weak evidence base that currently underpins the GAT approach. However, as discussed above, it is critical to note that this type of observational research is prone to bias, confounding, and lacks ability to distinguish treatment effects from placebo effects (Fanaroff et al., 2020; Pocock & Elbourne, 2000). Thus, it is unlikely to provide the rigorous empirical data that can convincingly demonstrate a causal relationship between treatment and outcome.

Further, there seems to be a problematic tension between the research and clinical agendas of CAGCs. GAT is being provided in a clinical environment that maximizes the placebo effect. This is the same environment in which the same clinicians are researching GAT’s efficacy. As previously discussed, while a placebo effect-enhancing environment may be appropriate for a clinical environment, it is far from an ideal treatment efficacy research environment, particularly when DBRCTs are not possible and RCTs are not undertaken. In the next section, I delve more deeply into exploring this issue. First, however, I will take a brief detour with an example that illustrates the risks when expert opinion and low-quality evidence are relied on as a basis for medical interventions.

## A Recent Example from Medical History of the Dangers of Medical Advice Based on Weak Evidence: The Iatrogenic Tragedy of Prone Infant Sleep Position and Sudden Infant Death Syndrome

Gender medicine clinicians and researchers have consistently stated that RCTs would be unethical (de Vries et al., 2011; Smith et al., 2001; Tollit et al., 2019). However, as Valenstein (1986) discussed in his study of the history of lobotomy, the ethics of implementing new treatments without a rigorous evidence base also need to be considered. The harm that can be done by well-intentioned, but erroneous medical advice based on prestigious physicians’ clinical judgment without an adequate evidence base can be illustrated by infant sleep position and sudden infant death syndrome (SIDS). Prior to the middle of the twentieth century, it was common practice for mothers to place infants on their backs to sleep (Högberg & Bergström, 2000). The influential pediatrician, Benjamin Spock, was an early advocate of the prone position (front sleeping) for infants. He recommended it in his popular book, Baby and Childcare, from the 1956 edition through until 1985 (Gilbert et al., 2005). This recommendation, that became widespread, was mainly based on clinical wisdom that such a position reduced risk of death from aspiration of vomit and had additional benefits such as decreased crying and reduced head flattening. Early research appeared to support this clinical advice. However, by the 1980s, more rigorous research demonstrated that the prone position increased risk of SIDS. Then medical advice gradually changed to strongly recommending infant supine (back) sleeping. A marked drop in SIDS rates followed. Several biases (e.g., the healthy adopter bias and observer bias) are thought to have contributed to the erroneous clinical belief that prone sleeping position was the safest position. It has been estimated that between the 1950s and the 1990s the infant prone sleeping advice, recommended by well-meaning clinicians and prestigious medical organizations, may have contributed to the deaths of tens of thousands of infants (Gilbert et al., 2005; Sperhake et al., 2018).

## Gender-Affirming Treatment for Youth with Gender Dysphoria: A Perfect Storm for Placebo Effect

The reader may ask: Why focus on GAT for GD youth? Is GAT any different from other contemporary medical treatments that also are not underpinned by rigorous evidence? I would reply—indeed, this is an issue in other areas of medicine. For example, the response rate in the placebo groups in antidepressant medication clinical trials is known to be high (Benedetti, 2021a). However, in contrast to GAT, we know this because there have been many RCTs comparing antidepressants to placebos. A recent review, that included placebo in the network meta-analysis, found that all the antidepressants under review were more efficacious than placebo in adults with major depressive disorder (Cipriani et al., 2018). This finding has been challenged by some who argue that the benefits of antidepressants beyond placebo effect seem to be minimal (Jakobsen et al., 2020). However, one of the key points to make is that placebo effect in antidepressant medication response is at least known about and discussed by many researchers, clinicians, and their patients (personal clinical experience), rather than not considered at all, as seems to be the situation to date for GAT for GD youth. Gender medicine clinicians and researchers might take note of a recent meta-analysis of antidepressants in pediatric populations, which recommended that the influence of placebo response needs to be considered in pediatric clinical trial design and implementation (Feeney et al., 2022). Furthermore, it seems particularly vital to consider the potential role of placebo effect in GAT outcomes because the stakes are high. Medical and surgical GAT, being given to vulnerable minors, lead to life-long medicalization and hold the risk of serious irreversible adverse impacts, such as sterility and impaired sexual function. Thus, we need strong evidence that they are as efficacious for critical mental health outcomes as claimed and that there are no less harmful alternatives.

In the field of GD youth medicine, there is a combination of features that seems to create a perfect storm setting for placebo effect. Thus, we have a population of vulnerable youth presenting with a condition, which has no objective diagnostic tests, and that is currently undergoing an unexplained rapid increase in prevalence and marked change in patient demographics. The treatment response is mainly based on patient-reported outcomes (yes, this can be the case for other conditions but remember we are considering the combination of features, not just a feature in isolation). Some clinicians, who may be affiliated with prestigious institutions, enthusiastically promote GAT, including on the media, social media, and alongside celebrity patients. Some make overstated claims about the strength of evidence and the certainty of benefits of GAT, including an emphasis on their “life-saving” qualities, and under-acknowledge the risks. Alternative psychosocial treatment approaches are sometimes denigrated as harmful and unethical conversion practices or as “doing nothing.” This combination of features increases the likelihood that there will be a complex interplay of heightened placebo and nocebo effects in this area of medicine, with significant implications for research and clinical practice. Some examples of these types of issues are now provided.

### Overstatement of the Certainty of Benefits and Under-Acknowledgment of Risks

Some professional organizations and leading GAT clinicians, in publicly available communications to GD youth, the public, and policy makers, appear to overstate the certainty of GAT’s benefits and provide inadequate discussions of risks (Clayton, 2022a; Cohen, 2021a, b; Olson-Kennedy, 2015, 2019; Telfer, 2019, 2021). For example, GATs have been described in such communications as “absolutely life-saving” (Olson-Kennedy, 2015) and being underpinned by “robust scientific research” (Telfer, 2019). It is notable that these same clinicians in their peer reviewed publications acknowledge the sparse empirical evidence with critical knowledge gaps (Olson-Kennedy et al., 2019), and the urgent need for more evidence for this relatively new treatment approach (Tollit et al., 2019). Thus, there seems to be a kind of Janus-faced narrative, with a placebo effect-enhancing face of overstated certainty/strong evidence of benefit displayed to GD youth, their families, and policy makers, and the more realistic face of uncertainty/lack of evidence turned toward peer reviewers and the research community. Of note, several publications in the peer review literature that have made overstated claims about GAT have recently required correction (Bränström & Pachankis, 2020; Pang et al., 2021; Zwickl et al., 2021).

### The Dangers of an Exaggerated Suicide Narrative

A specific issue that is important to discuss is the repeated claims that GD youth are at high suicide risk and that GAT reduces this risk. Parents report being told by clinicians that their child will suicide if a trans identity is not affirmed (Jude, 2021). Clinicians’ public statements also indicate this message is being given, or at least implied, to parents and young people (Cohen, 2021b; Marchiano, 2017). A recent editorial in *The Lancet* stated puberty blockers reduce suicidality and to remove access to them was to “deny” life (The Lancet, 2021). However, there is no robust empirical evidence that puberty blockers reduce suicidality or suicide rates (Biggs, 2020; Clayton et al., 2021). The authors of the paper, that was the basis for *The Lancet’s* claim, subsequently clarified that they were not making any causal claims that puberty blockers decreased suicidality (Rew et al., 2021). Another paper, claiming to have found that barriers to gender-affirming care was associated with suicidality, had to withdraw this claim in a significant correction to their article (Zwickl et al., 2021).

Furthermore, the suicidality of GD youth presenting at CAGCs, while markedly higher than non-referred samples, has been reported to be relatively similar to that of youth referred to generic child and adolescent mental health services (Carmichael, 2017; de Graaf et al., 2022; Levine et al., 2022). A recent study reported that 13.4% of one large gender clinic’s referrals were assessed as high suicide risk (Dahlgren Allen et al., 2021). This is much less than conveyed by the often cited 50% suicide attempt figure for trans youth (Tollit et al., 2019). A recent analysis found that, although higher than population rates, transgender youth suicide (at England’s CAGS) was still rare, at an estimated 0.03% (Biggs, 2022).

Of course, any elevated suicidality and suicide risk is of concern, and any at risk adolescent should be carefully assessed and managed by expert mental health professionals. However, an excessive focus on an exaggerated suicide risk narrative by clinicians and the media may create a damaging nocebo effect (e.g., a “self-fulfilling prophecy” effect) whereby suicidality in these vulnerable youths may be further exacerbated (Biggs, 2022; Carmichael, 2017). This type of risk has been discussed in other similar situations involving youth (Abrutyn et al., 2020; Canetto et al., 2021; Shain & AAP COMMITTEE ON ADOLESCENCE, 2016).

### An Excessively Negative Portrayal of the Previous Standard and Current Alternative Treatment Options

Clinicians and groups advocating for GAT tend toward framing any non-affirming treatment approaches as harmful, ineffective, and unethical, and sometimes equate psychotherapeutic approaches with conversion practices (Ashley, 2022). However, others argue that there are a range of contemporary therapeutic approaches which are not “affirmative,” but neither are they conversion practices (D’Angelo et al., 2021). Such approaches can include: Careful assessment and diagnostic formulation, appropriate treatment of co-existing psychological conditions, supportive and educative individual/family psychological care, group therapy, developmentally informed gender exploratory psychotherapy, trauma-informed psychotherapy, and a non-promotion of early childhood social transition (sometimes labeled under the umbrella term of “watchful-waiting,” which should not be interpreted as “doing nothing”) (D’Angelo et al., 2021; de Vries & Cohen-Kettenis, 2012; Hakeem, 2012; Kozlowska et al., 2021; Lemma, 2021).

It is important to note that psychotherapeutic approaches for this group of patients are also based on limited evidence. More research into their efficacy is required. One critical consideration here seems to be that ethical psychological approaches do not hold the same adverse risk profiles as do the hormonal and surgical treatments (Baron & Dierckxsens, 2021).

Recently, two Scandinavian youth gender services have drawn similar conclusions and instigated a more cautious approach to hormonal treatments for GD minors, placing a higher emphasis on psychological care (Kaltiala-Heino, 2022; Socialstyrelsen, 2022). Furthermore, in England, the Cass Review into the country’s youth gender services has released its interim report (Cass, 2022). In response, the National Health Service’s “Interim Service Specification” for GD youth specialist services has specified that the primary intervention for youth will be psychosocial support and psychological interventions. A cautious approach to social transition is recommended and puberty blockers will only be available in the context of a formal research protocol (National Health Service, 2022).

Given all this, it is hard to accept the claims that GAT is prima facie the best treatment model for today’s cohort of GD youth. Furthermore, the unwarranted negative portrayal of contemporary psychotherapeutic approaches likely creates nocebo effects and undermines the possibility of providing such ethical care to GD youth (Kozlowska et al., 2021).

### Clinicians’ Media and Social Media Promotion of Gender Affirmative Treatment

There is intense media and social media coverage of “trans youth” issues. Some surgeons are promoting their gender-affirming surgeries on social media platforms that are popular with young adolescents (Ault, 2022). Some clinicians encourage the celebratory media coverage of GAT, stating it may empower young trans people to seek GAT (Pang et al., 2020). They largely dismiss concerns that the identified association between positive media stories and increased referral rates to CAGCs may be indicative of a social contagion phenomenon. This is despite the reports of the sudden emergence of gender dysphoria, especially in adolescence, and its association with social influence (Kaltiala-Heino, 2022; Littman, 2018, 2021; Marchiano, 2017). Gender clinicians also condemn and have attempted to prevent what they consider as excessively negative media coverage of GAT (although others judge it as reasonable and balanced) (Australian Press Council, 2021; Pang et al., 2022). These clinicians are likely correct that critical media coverage of GAT could negatively impact referrals to gender clinics and might upset some patients. However, a deliberate strategy of promoting an unbalanced celebratory GAT narrative through the media and social media could contribute to social contagion and placebo effects.

What is the right balance? The Australian Press Council’s judgment on a clinician’s complaints about what she considered as excessively negative press coverage may, arguably, provide an example of some balance on these matters. Of note, while some of the complaints were upheld, many were not and it was judged that “even medical treatment accepted as appropriate by a specialist part of the medical profession is open to examination and criticism…needs to be debated…and was sufficiently justified in the public interest” (Australian Press Council, 2021).

### The Exclusive Promotion of Gender-Affirming Treatments within Child and Adolescent Gender Clinics

There is indication of an unbalanced promotion of a celebratory GAT narrative occurring within CAGCs, where, simultaneously, there is a deep enmeshment of the clinical, advocacy, and research agendas. This has already partly been discussed in the sections above, but one detailed example is provided. The Trans20 study is a prospective cohort study based on children and adolescents seen at Melbourne’s Royal Children’s Hospital Gender Clinic (RCHGC) which provides a GAT model of care (Telfer et al., 2018; Tollit et al., 2019). It is important to highlight that this study’s human research ethics committee (HREC) approval was not for the treatment approach, which was implemented as routine clinical care, rather it was for matters such as collection and storage of data, and longitudinal follow-up of discharged patients.

In 2019, an amended HREC approval was granted, allowing a “newsletter blog” to be sent to patients and families with the aim of improving patient engagement with the study. This change was described as raising no new ethical issues. This “first ever” newsletter asked for help with the Trans20 survey completion (Royal Children’s Hospital, 2019). This research request was placed amid positive accounts of the service and its patients. For example, following attendance at the clinic’s single session assessment triage (SSNac) young people were described as feeling “empowered…and more likely to start living as their preferred gender,” and having improvements in mental health and quality of life. A colorful diagram showed the increased rates of social transition that followed SSNac attendance, and the section concluded “Hopefully the improvements after SSNac are a taste of things to come!” One pro-GAT parent/carer support network, that also fundraises for the RCHGC, was spotlighted. There was a “lived experience” piece in which a well-known transitioned, now young adult, patient was pictured receiving an award. This patient provided a personal testimony of the clinic’s medical director: She “will always be one of my biggest heroes…an incredible person: Intelligent, compassionate and strong.”

This newsletter’s communication raises much to think about. The point I want to make here is that sandwiching the requests for a research survey completion between celebratory accounts of the clinic seems likely to magnify the impact of bias and placebo effect on research outcome findings. For example, consider the likely impact on patient bias (patients wanting to please the clinician by giving positive reports), response bias (patients with positive experiences of the clinic more likely to complete the surveys), social learning/contagion, prestige suggestion, and the Hawthorne phenomenon. Furthermore, consider this newsletter as part of the whole therapeutic ritual, enhancing the psychological and neurobiological placebo mechanisms. Apart from this research impact, we can also wonder whether such a newsletter is ideal clinical practice. In my opinion, there are problems. Think, for example, of the young GD patient who may be hesitant to transition. Where is the celebration of this young person’s choices? Communications from the clinic, such as this newsletter, may contribute to feelings that, unless he/she transitions, he/she lacks courage (having not been “empowered”) and that he/she will never be an award-winning celebrated patient. This may act as a covert form of pressure on patients to transition or, for those who do not, act as a nocebo effect negatively impacting their psychological outcomes.

## Where to From Here?

There are no easy solutions to the complex research and clinical issues presented in this Letter. Here, I present a few ideas to stimulate discussion. The first step would seem to be more professional awareness and debate. Independent reviews by expert clinicians and methodologists, not currently involved in clinical practice and research in this area (thus, having some emotional distance and minimizing intellectual conflict risk), could helpfully advise further research and clinical strategies. England’s Cass Review is an example of this type of approach (Cass, 2022).

Clinicians should also make measured and honest statements to patients, families, policy makers, and the public about the evidence for GAT’s benefits. Placebo effects could also be noted in the limitations section of any research papers. In addition, in public discourse, the media and clinicians could present not only celebratory transition stories, but also: Realistic positive stories of those with gender dysphoria who have decided not to transition or have delayed transition until maturity; accounts of patients who have benefitted from ethical psychological approaches; and accounts of those who have had negative transition experiences. Detransition, regret, and harm from transition should be acknowledged and publicized as a significant risk. A recent paper detailing the elements of a comprehensive informed consent process is timely and important (Levine et al., 2022). However, while a comprehensive informed consent process is vital, it does not address the issue of how the whole ethos of a clinic and the media/social media milieu may act to influence young patients and their families and undermine the capacity for true informed consent.

## Conclusion

In conclusion, this Letter has noted that although GAT for GD youth lacks a rigorous evidence base, it is undertaken as routine medical treatment in a strongly placebo effect enhancing environment. It is within this environment that research into its effectiveness is being undertaken. One consideration raised by this relates to clinical practice: When does such a strongly placebo effect enhancing environment meet optimal clinical practice standards? When, if at all, does it veer into the territory of unethical practice that involves deception and undue influence? This Letter has also highlighted that such a placebo effect enhancing environment presents grave problems for research (particularly non-DBRCT research). It seems unlikely that the current research being undertaken in this field will be able to untangle benefits that are due to the placebo effect from those due to the interventions’ specific effectiveness. Thus, especially given the adverse risk profile of the hormonal and surgical interventions, it may be that yet again well-intentioned physicians are engaging in medical practices that cause more harm than benefit (Clayton, 2022b). The research and clinical conundrums presented in this Letter have no easy answers. However, as a first step, there is an urgent need for more awareness of the placebo effect and for rigorous and thoughtful debate over how best to proceed in research and clinical practice in this area of medicine.
